# Quantification of Growth of *Campylobacter* and Extended Spectrum β-Lactamase Producing Bacteria Sheds Light on Black Box of Enrichment Procedures

**DOI:** 10.3389/fmicb.2016.01430

**Published:** 2016-09-12

**Authors:** Wilma C. Hazeleger, Wilma F. Jacobs-Reitsma, Heidy M. W. den Besten

**Affiliations:** ^1^Laboratory of Food Microbiology, Wageningen UniversityWageningen, Netherlands; ^2^National Institute for Public Health and the EnvironmentBilthoven, Netherlands

**Keywords:** ESBL, pre-enrichment, ISO 10272-1, Bolton broth, Preston broth, clavulanic acid, competition, inhibition

## Abstract

*Campylobacter* is well recognized as the leading cause of bacterial foodborne diarrheal disease worldwide, and is routinely found in meat originating from poultry, sheep, pigs, and cattle. Effective monitoring of *Campylobacter* contamination is dependent on the availability of reliable detection methods. The method of the International Organization for Standardization for the detection of *Campylobacter* spp. in food (ISO 10272-1:2006) recommends the use of Bolton broth (BB) as selective enrichment medium, including a pre-enrichment step of 4–6 h at 37°C to revive sublethally damaged cells prior to incubation for 2 days at 41.5°C. Recently the presence of abundantly growing extended spectrum β-lactamase producing Enterobacteriaceae (ESBL bacteria) has become one of the most important factors that interfere with the isolation of *Campylobacter*, resulting in false-negative detection. However, detailed growth dynamics of *Campylobacter* and its competitors remain unclear, where these would provide a solid base for further improvement of the enrichment procedure for *Campylobacter*. Other enrichment broths, such as Preston broth (PB) and BB plus clavulanic acid (BBc) have been suggested to inhibit competitive flora. Therefore, these different broths were used as enrichments to measure the growth kinetics of several strains of *Campylobacter jejuni* and ESBL bacteria separately, in co-culture and of strains in chicken samples. The maximum cell numbers and often the growth rates of *Campylobacter* in mixed culture with ESBL bacteria were significantly lower than in single cultures, indicating severe suppression of *Campylobacter* by ESBL bacteria, also in naturally contaminated samples. PB and BBc successfully diminished ESBL bacteria and might therefore be a better choice as enrichment medium in possibly ESBL-bacteria contaminated samples. The efficacy of a pre-enrichment step in the BB ISO-procedure was not supported for cold-stressed and non-stressed cells. Therefore, omission of this step (4–6 h at 37°C) might be advised to obtain a less troublesome protocol.

## Introduction

Campylobacteriosis is the most commonly reported zoonosis in the European Union showing an increasing trend over the period of 2008–2014, and the occurrence of thermotolerant *Campylobacter* in broiler meat remains high at 38.4% in 2014 (European Food Safety Authority [EFSA] and European Centre for Disease Prevention and Control [ECDC], 2015). Concomitantly, broilers are often contaminated with extended spectrum β-lactamase producing Enterobacteriaceae (ESBL bacteria; Bortolaia et al., 2010; Depoorter et al., 2012; Dierikx et al., 2013; Kawamura et al., 2014; Olsen et al., 2014). The presence of these ESBL bacteria in food has become an important factor interfering with the isolation of *Campylobacter*, resulting in false-negative detection, since abundantly growing ESBL bacteria hamper the isolation of *Campylobacter* colonies (Jasson et al., 2009).

The protocol of the International Organization for Standardization (ISO) for detection of thermotolerant *Campylobacter* spp. in food and animal feeding stuffs (ISO, 2006), describes the use of Bolton broth (BB) which is mixed 10:1 with the food sample including a 4–6 h pre-enrichment step at 37°C to resuscitate sublethally damaged cells before further enrichment is done at 41.5°C for 2 days. After enrichment, campylobacters are isolated on modified charcoal-cefoperazone-deoxycholate agar (mCCDA) and a second selective medium, with a principle different from mCCDA. The antibiotics in BB and mCCDA do not inhibit the growth of ESBL bacteria (Jasson et al., 2009), therefore the selectivity of the media is diminished. Nonetheless, detailed growth dynamics of *Campylobacter* and its competitors during enrichment remain unclear, and these would provide a good starting point for developing a proper enrichment procedure for *Campylobacter*. Furthermore, strong scientific evidence for better isolation of the pathogen using a pre-enrichment step at 37°C is scarce (Humphrey, 1986, 1989).

Therefore, in this study, growth kinetics of *Campylobacter* and ESBL bacteria were determined during the enrichment procedure in BB and also in previously suggested alternative enrichment broths, such as Preston broth (PB; Scotter et al., 1993; Uyttendaele and Debevere, 1996) and BB plus clavulanic acid (BBc; Moran et al., 2011). Single strains and mixed cultures of *Campylobacter* and ESBL bacteria were tested and also naturally contaminated samples were examined. To create sublethally damaged cells, naturally contaminated chicken samples and inoculated chicken samples were cooled and/or frozen previously to the enrichment procedures to determine the effect of the pre-enrichment step at 37°C on the recovery of *Campylobacter*.

## Materials and Methods

### Bacterial Strains and Preparation of Stationary Phase Cultures

*Campylobacter jejuni* ATCC 33560 (= NCTC 11351), which is indicated as suitable control strain (ISO, 2006), *C. jejuni* LU 160891 (Wageningen University; isolate from chicken filet), *Campylobacter coli* WCDM 00004, a strain advised for performance testing (ISO, 2015), and *Escherichia coli* ESBL strains RIVM 2 and RIVM 3 (National Institute for Public Health and the Environment; isolates from chicken filet) were used as single cultures and as *Campylobacter* and ESBL–*E. coli* mixed cultures. *Campylobacter* stock cultures were grown in Heart Infusion broth (HI, Becton Dickinson) for 48 h at 37°C, then supplemented with 15% glycerol and stored at -80°C. *E. coli* stocks were cultured in Brain Heart Infusion broth (BHI, Becton Dickinson) for 24 h at 37°C, then supplemented with 15% glycerol and stored at -80°C as well. To obtain precultures for the growth experiments, *C. jejuni* was plated from the -80°C vials onto Columbia agar base (CAB, Oxoid, supplemented with 5% (v/v) lysed sheep blood (BioTrading Benelux B.V. Mijdrecht, Netherlands)) and grown for 48 h at 37°C, whereas ESBL *E. coli* was plated onto BHI agar or tryptone soya agar (TSA, Oxoid) and grown for 24 h at 37°C. Subsequently, single colonies were resuspended in HI and BHI for *C. jejuni* and *E. coli*, respectively, and cultured at 37°C for, respectively, 48 and 24 h to obtain stationary phase cultures. Cell concentrations were determined by plating appropriate dilutions on CAB for *Campylobacter* and on TSA for ESBL *E. coli*. *Campylobacter* was cultured under micro-aerobic conditions (5% O_2_, 10% CO_2_, 85% N_2_) in flushed jars (Anoxomat WS9000, Mart Microbiology, Drachten, Netherlands) unless stated otherwise.

### Pretreatment of Chicken Samples

To determine the effect of a pre-enrichment step for 4–6 h at 37°C on the growth of sublethally damaged cells, inoculated chicken samples were stored at 4°C or -20°C, to mimic the situation in practice where chicken samples are purchased in cooled, respectively, frozen state. For that, chicken skin samples (5 g, confirmed previously to be *Campylobacter*-free, using ISO 10271-1 (ISO, 2015), were kindly provided by Johan Roelofs, Plukon B.V., Wezep, Netherlands). The samples were stored at -20°C, thawed before use at 21°C for a maximum of 30 min and then inoculated with about 0.5 mL of diluted 48 h cultures [10^2^ to 10^4^ colony forming units (CFU) per 5 g chicken skin sample] of *C. jejuni* or *C. coli* and/or 24 h cultures of ESBL *E. coli* and subsequently stored in stomacher bags (Antonides, Oosterzee, Netherlands) for 60 h at 4°C or -20°C. Choices of inoculation levels were aiming for similar starting levels of *Campylobacter* and ESBL bacteria after the cooling or freezing treatment which were determined in separate experiments, where the reduction in cell numbers on chicken stored at 4°C and -20°C was quantified (data not shown). Frozen samples were thawed for 30 min at 21°C and cooled samples were allowed to reach room temperature for 5 min before chicken juice was prepared (see Preparation of Chicken Juice) and then enrichment procedures were started.

### Preparation of Chicken Juice

To be able to take regular samples in time without disturbing the micro-aerobic conditions, growth curves were made in infusion bottles (100 mL), sealed with a thick (1 cm) rubber stopper and secured by an aluminum cap. Chicken juice of inoculated chicken skin (*n* = 12), was prepared to allow adding of chicken product to the broth using a syringe and at the same time to recover as much of the present micro flora as possible to meet the ISO-procedure (ISO, 2006). Chicken skin juices were made by adding chicken skin at a 1:1 ratio to peptone physiological salt [PPS; 0.9% NaCl and 0.1 % peptone (Oxoid)] in a filter stomacher bag. The bag was massaged by hand for 2 min, and further homogenized for 30 s in a Pulsifier 100E (Microgen Bioproducts, Camberley, UK). Juice from the inoculated chicken skin was then immediately used in the enrichment procedures (see Measuring Growth Dynamics).

To examine naturally contaminated chicken liver, chicken juices were prepared similarly to the inoculated chicken skin as described above. For chicken wings, the same procedure was followed, except the samples were mixed in PPS at a 2:1 ratio. To confirm and quantify presence of *Campylobacter* and ESBL bacteria, 1 mL of juice was spread onto three Campyfood agar plates (CFA, bioMérieux) or Rapid’ *Campylobacter* agar plates (RCA, Bio-Rad) and onto Brilliance ESBL plates (Oxoid), respectively. *Campylobacter* plates were incubated for 48 h at 41.5°C (micro-aerobic conditions) after which confirmation was done microscopically and using a Latex agglutination test for *Campylobacter* (Microgen Bioproducts). Brilliance ESBL plates were incubated for 24 h at 37°C. All chicken juices were stored for 2–3 days in 50 mL Greiner tubes at 4°C until the results of the plating showed presence of *Campylobacter* and ESBL bacteria and these juices (*n* = 26) were then directly used in the enrichment procedures (see Measuring Growth Dynamics). For ESBL bacteria all colonies were counted on the Brilliance ESBL plates and no distinction was made between *E. coli* or other ESBL containing bacteria.

### Measuring Growth Dynamics

Infusion bottles were filled with 45 mL of enrichment medium. After that, either 5 mL of diluted stationary phase cultures (to mimic a *C. jejuni* concentration of 2–3 log CFU g^-1^ chicken (European Food Safety Authority [EFSA], 2011), resulting in a starting concentration in the enrichment broth of about 1–2 log_10_ CFU mL^-1^), or 5 mL of chicken juice were added and the head space was flushed for 2 min with a gas-mixture of 5% O_2_, 10% CO_2_, and 85% N_2_ by a home-made gas-flushing device using syringes to puncture the rubber stopper of the bottles. BB (Oxoid) with selective supplement (Oxoid SR0208E) and 5% of sterile lysed horse blood (Oxoid) or sheep blood (Biotrading) was used as enrichment medium. Also alternative enrichment broths were used; to obtain BBc, 2 mg L^-1^ (end concentration) potassium clavulanate (Sigma-Aldrich) was added to BB (Moran et al., 2011). PB was prepared as Nutrient Broth No. 2 (Oxoid) with Preston *Campylobacter* Selective Supplement (Oxoid SR0204), *Campylobacter* Growth Supplement (Oxoid), and 5% of lysed horse blood. Inoculated infusion bottles were incubated in water baths set at 37°C (4–6 h) and 41.5°C (up to 48 h).

At regular time intervals, 1 mL samples were taken from the bottles using a syringe and after every second sample, bottles were flushed again with the appropriate gas. Samples were immediately diluted and plated onto CFA, RCA, or mCCDA (Oxoid, supplemented with Oxoid SR155E) for counting *Campylobacter* and onto Brilliance ESBL agar for ESBL bacteria, and incubated as described in Section “Preparation of Chicken Juice.” In order to prevent swarming of *Campylobacter* colonies, all plates for enumeration were dried in a 41.5°C incubator for 15–20 min before plating. At least two biologically independent reproductions per strain or strain combination were performed on different days in all enrichment media.

### Data Analysis

Plate counts were transformed to log_10_ CFU mL^-1^ and growth curves were constructed using Microsoft Excel 2010 and counts were fitted with the modified Gompertz Model (Zwietering et al., 1990) using the Solver add-in of Excel. The analysis was verified using TableCurve 2D V5.01 and a *t*-test was used to examine statistical significance in the growth parameters λ (lag phase; h) and μ (maximum growth rate; log_10_ h^-1^) of the microorganisms at the different conditions (*P* < 0.05).

## Results

### Single and Mixed Cultures

**FIGURE 1:**
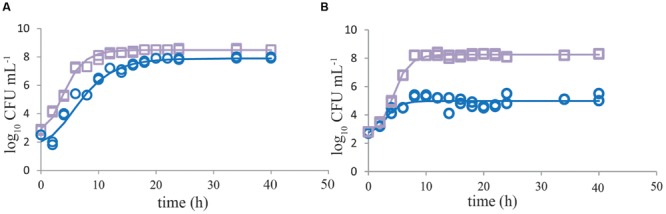
**Growth of *Campylobacter jejuni* is severely inhibited when cocultured with ESBL *E. coli.*** Representative growth curves (*n* = 2) of *C. jejuni* ATCC 33560 (blue circles) and ESBL *E. coli* RIVM 2 (purple squares) in Bolton broth (48 h 41.5°C) when cultured as single cultures **(A)** and in co-cultures **(B)**. Strains were precultured to stationary phase and subsequently inoculated in enrichment broth. The lines are curves fitted with the modified Gompertz model (Zwietering et al., 1990). Detection limit is 1 CFU mL^-1^.

**FIGURE 2:**
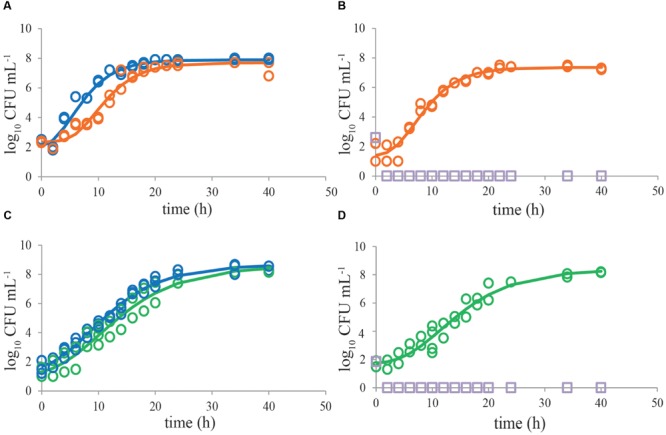
**Bolton broth plus clavulanic acid and Preston broth inhibit growth of ESBL *E. coli* RIVM 2 whereas *Campylobacter jejuni* ATCC 33560 exhibits good growth in all media.** Representative growth curves (*n* = 2) of *C. jejuni* ATCC 33560 as single cultures in Bolton broth (48 h 41.5°C; blue circles, **A,C**), in Bolton broth plus clavulanic acid (orange circles, **A**), and in Preston broth (green circles, **C**). Representative growth curves (*n* = 2) of mixed cultures of *C. jejuni* ATCC 33560 (orange, respectively, green) and ESBL *E. coli* RIVM 2 (purple squares) in Bolton broth plus clavulanic acid **(B)** and Preston broth **(D)**. Strains were precultured to stationary phase and subsequently inoculated in the respective enrichment broths. The lines are curves fitted with the modified Gompertz model (Zwietering et al., 1990). Detection limit is 1 CFU mL^-1^.

**FIGURE 3:**
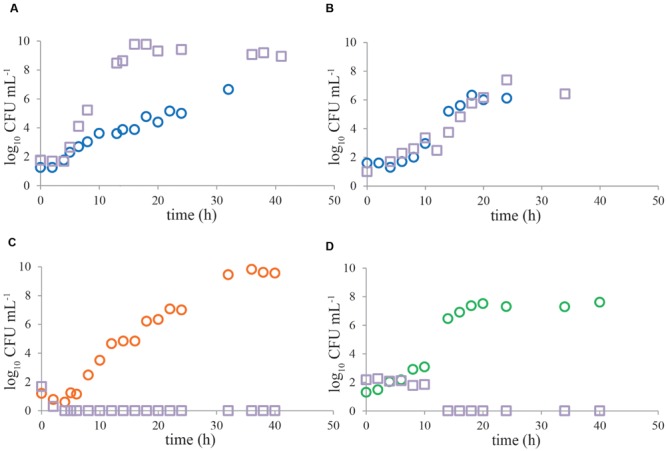
**Representative growth curves of *Campylobacter* and ESBL-producing bacteria in naturally contaminated chicken samples.** Enrichment was done in Bolton broth **(A,B)**, Bolton broth plus clavulanic acid **(C)** and Preston broth **(D)**. In most of the cases (*n* = 23) in Bolton broth, *Campylobacter* (circles) was outcompeted by ESBLs (squares) **(A)**, however, in three cases, growth kinetics were similar **(B)**. The alternative enrichment broths Bolton broth plus clavulanic acid (*n* = 11) and Preston broth (*n* = 7) always completely inhibited growth of ESBL bacteria **(C,D)**. Panels **(A,C)** are chicken wing samples, panels **(B,D)** are chicken liver samples. Detection limit is 1 CFU mL^-1^.

Growth of single *C. jejuni* strains in BB showed that using a start inoculum of 2 log_10_ CFU mL^-1^, the maximum level (8 log_10_ CFU mL^-1^) was reached after about 20 h (**Figure 1A**). ESBL *E. coli* showed higher growth rate, and reached stationary growth phase already after 10–12 h of incubation (**Figure 1A**). When both strains were cultured together, growth of ESBL *E. coli* was comparable to single culture conditions. *C. jejuni*, however, showed severe growth reduction in mixed culture with ESBL *E. coli* (**Figure 1B**). When ESBL *E. coli* reached the stationary phase, or just before that, growth of *C. jejuni* ceased and remained at a maximum level of 4–6 log_10_ CFU mL^-1^. Due to the limited number of data points in the beginning of these curves, lag phases could not be determined accurately but seemed to be non-significant in most cases. In general, growth rates of *C. jejuni* in BBc (**Figures 2A,B**) and PB (**Figures 2C,D**) were similar to growth in BB (*P* > 0.05), although some lag time was observed in BBc. However, BBc and PB successfully inhibited growth of ESBL *E. coli*, both in single cultures (data not shown) and in co-cultures with *C. jejuni* (**Figures 2B,D**). Similar results were found for all tested *C. jejuni* and ESBL *E. coli* strains. Since no difference was observed in data with and without pre-enrichment at 37°C, only graphs without pre-enrichment are shown (see Effect of Pre-enrichment) for the results described in **Figures 1–3**.

### Naturally Contaminated Samples

Growth characteristics of *Campylobacter* and ESBL bacteria from most naturally contaminated samples show similar trends as mixed cultures (**Figure 3**), even though the naturally contaminated samples were stored refrigerated before enrichment. In BB, *Campylobacter* was mostly outcompeted (**Figure 3A**) by ESBL bacteria (*n* = 23). However, in 12% of the cases (*n* = 3), the pathogen was able to grow to similar levels as ESBLs (**Figure 3B**). The alternative enrichment broths PB (*n* = 7) and BBc (*n* = 11) always completely inhibited growth of ESBL bacteria (**Figures 3C,D**), comparable to the situation in laboratory strains (**Figure 2**).

### Effect of Pre-enrichment

**FIGURE 4:**
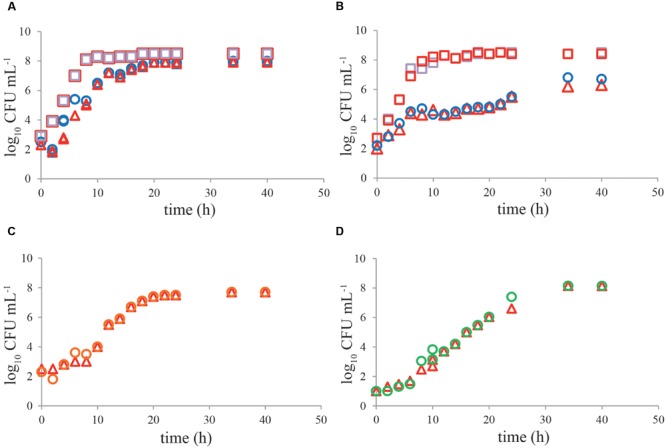
**No effect of pre-enrichment on growth of *Campylobacter jejuni* ATCC 33560 and ESBL *E. coli* RIVM 2 in Bolton broth, Bolton broth with clavulanic acid, and Preston broth.** Representative growth curves (*n* = 2) of *C. jejuni* ATCC 33560 (circles and triangles) in Bolton broth **(A,B)**, Bolton broth plus clavulanic acid **(C)**, and Preston broth **(D)** as single culture **(A,C,D)** with (4 h 37°C, 44 h 41.5°C; red triangles) and without (48 h 41.5°C; circles) pre-enrichment step. Growth of ESBL *E. coli* RIVM 2 (squares) with (4 h 37°C, 44 h 41.5°C; red squares) and without (48 h 41.5°C; purple squares) pre-enrichment step in Bolton broth as single culture **(A)** and in mixed culture **(B)** with *C. jejuni* ATCC 33560. Strains were precultured to stationary phase and subsequently inoculated in the respective enrichment broths. Detection limit is 1 CFU mL^-1^.

**FIGURE 5:**
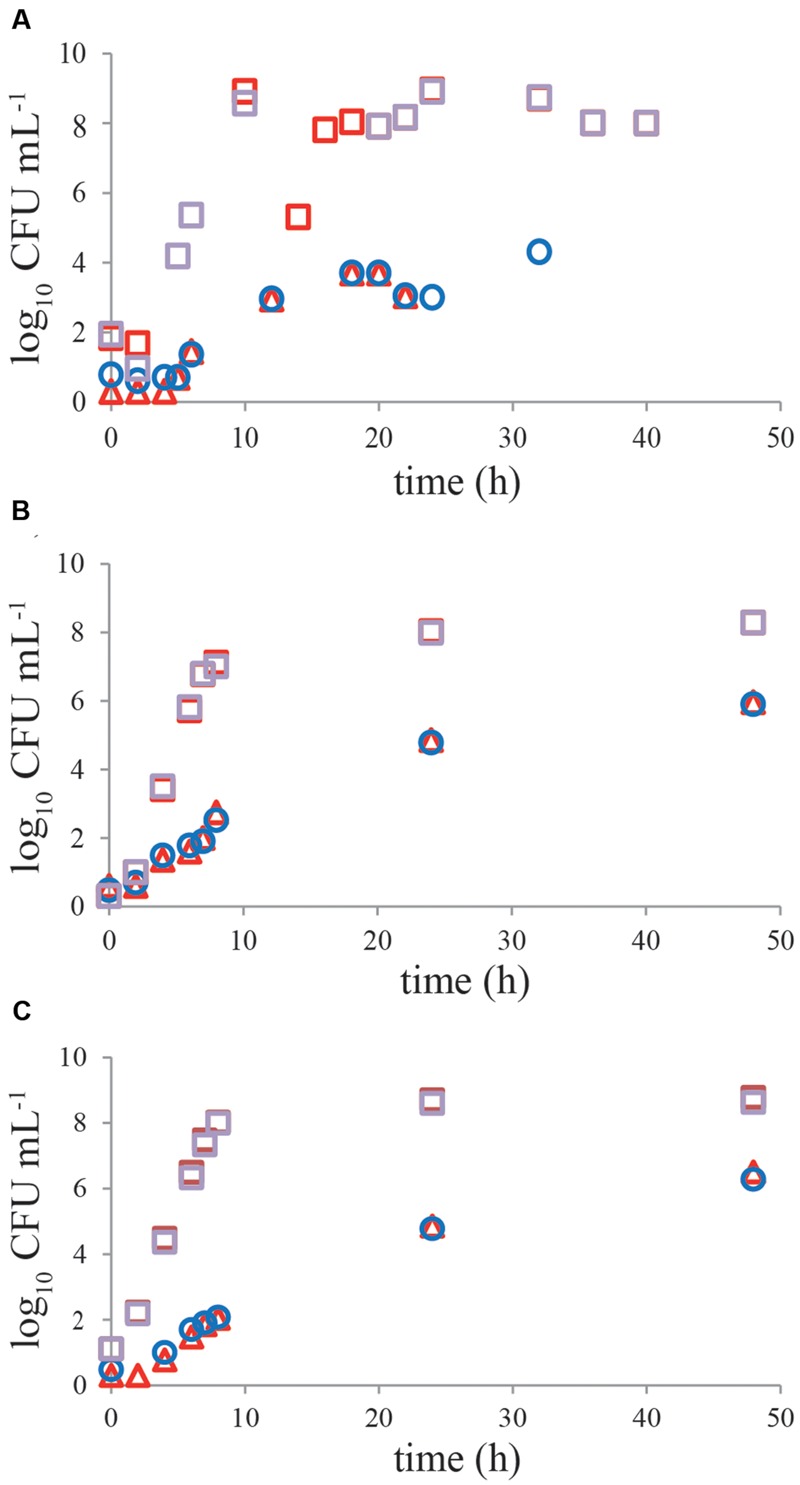
**A pre-enrichment step of 4–6 h at 37°C does not influence growth of cold-stressed *Campylobacter* and ESBLs from naturally contaminated or inoculated chicken products.** Representative growth curves of *Campylobacter* (circles and triangles) and ESBL-producing bacteria (squares) in Bolton broth. **(A)** Naturally contaminated chicken wing; **(B)** inoculated chicken neck skin pretreated for 60 h at 4°C; **(C)** inoculated chicken neck skin pretreated for 60 h at -20°C. Chicken samples were incubated with (4–6 h 37°C, 44 h 41.5°C; red symbols) and without (48 h 41.5°C; blue, purple symbols) pre-enrichment step in Bolton broth. Detection limit is 1 CFU mL^-1^.

No significant difference (*P* > 0.05) was observed in growth kinetics of *C. jejuni* or ESBL *E. coli* grown with and without a pre-enrichment incubation step for 4–6 h at 37°C, in single cultures or mixed cultures in BB (**Figures 4A,B**). For *C. jejuni* no effect of pre-enrichment was found in BBc (**Figure 4C**) or PB (**Figure 4D**) either. Similar results were found for all tested *Campylobacter* and ESBL *E. coli* strains. Growth characteristics of the cold-stressed bacteria from naturally contaminated chicken samples showed similar results for BB, where no effect of the pre-enrichment step at 37°C was observed either (**Figure 5A**). Cooling (60 h at 4°C) and freezing (60 h at -20°C) of artificially contaminated chicken neck skin samples resulted in, respectively, a 0.3 log_10_ and 1.5 log_10_ average reduction in *Campylobacter* numbers (calculated in CFU mL^-1^ BB). ESBL *bacteria* were not reduced (max 0.1 log_10_ reduction) at 4°C and 0.1–0.3 log_10_ reduced after freezing. After applying these stress conditions, a pre-enrichment step of 6 h at 37°C did not lead to better growth of *C. jejuni, C. coli* or ESBL bacteria compared to enrichment starting immediately at 41.5°C for cold stored samples or frozen samples (**Figures 5B,C**, respectively).

## Discussion

Selectivity of BB is based on the addition of four antibiotics; vancomycin, trimethoprim, amphotericin B, and cefoperazone, where the latter two are also used in the isolation plate (mCCDA) in the ISO-protocol for detection of *Campylobacter* (ISO, 2006). Cefoperazone is an antibiotic belonging to the group of third generation cephalosporins, and the β-lactam ring in this antibiotic is easily hydrolyzed by ESBL-containing organisms, rendering them insensitive to the selective compound (Chong et al., 2011). This fact, in combination with the increasing numbers of ESBL bacteria in chicken products (Costa et al., 2010; Overdevest et al., 2011; Dierikx et al., 2013), results in reduced isolation efficacy of *Campylobacter*, due to overgrowth of ESBL bacteria (Jasson et al., 2009). In the present study, the proliferation during enrichment was followed for both *Campylobacter* and ESBL bacteria. ESBL bacteria showed significantly higher growth rates in BB than the target organism (*P* < 0.05). In mixed cultures with *Campylobacter*, this resulted also in higher maximum cell numbers for ESBL bacteria, where the ESBL bacteria probably profit from their higher growth rate and the growth of *Campylobacter* ceased just before, or when the ESBL bacteria entered the stationary phase in growth. The observed differences in maximum cell numbers in mixed growth cultures, showed a 2–4 log_10_ CFU reduction of the target organism compared to single cultures. This clearly explains the difficulties in recognizing *Campylobacter* colonies on an mCCDA plate if 100- to 10,000-fold ESBL bacterial colonies are present, keeping in mind that growth of ESBL bacteria on mCCDA is not hindered either (data not shown). In a limited number of the cases, however, *Campylobacter* did grow to similar levels as ESBL bacteria, apparently these strains were not too much affected by ESBL bacteria, which can be partly explained by a very low initial number of ESBL bacteria in some of the naturally contaminated samples or ESBL bacteria with different growth characteristics. The ratio in numbers of *Campylobacter* and ESBL bacteria and species or type of both microorganisms may also be contributing factors to the growth dynamics during enrichment procedures. In this study, starting levels of around 10–100 CFU mL^-1^ were chosen in the enrichment broths, to aim for realistic levels reported in chicken of about 10–50 CFU g^-1^ product for ESBL bacteria (Cohen Stuart et al., 2012) and 10–1000 CFU g^-1^ for *Campylobacter* (European Food Safety Authority [EFSA], 2011).

To reduce growth of ESBL bacteria, β-lactamase inhibitors have been suggested (Payne et al., 1994), for instance tazobactam in the isolation medium mCCDA, showing good repression of ESBL bacteria (Smith et al., 2015). To increase selectivity of the enrichment in BB, addition of potassium clavulanate, another β-lactamase inhibitor, was suggested and examined (Moran et al., 2011; Chon et al., 2013a). In the current study, potassium clavulanate indeed inhibited the growth of ESBL bacteria in single and mixed cultures and also in naturally contaminated samples. The growth was not only inhibited, numbers of ESBL bacteria were even reduced to below the detection limit mostly within 4 h showing that selectivity of the medium was efficiently restored.

As alternative enrichment broth, PB has previously been described providing good selectivity against non-target flora in the enrichment procedure of *Campylobacter* (Bolton and Robertson, 1982; Uyttendaele and Debevere, 1996; Jasson et al., 2009; Habib et al., 2011; Ugarte-Ruiz et al., 2012) with selective components polymyxin B, rifampicin, trimethoprim, and cycloheximide/amphotericin B. Polymyxin B is probably the component that inhibits the ESBL bacteria since it has been shown to be active against most Gram-negative bacteria (Bolton and Robertson, 1982). Chon et al. (2013b) used polymyxin B in enrichment broth with cefoperazone, and restored selectivity in that way. Some studies have, however, shown that PB may inhibit growth of some *Campylobacter* strains as well, resulting in false negative outcomes (Baylis et al., 2000; Paulsen et al., 2005), especially for *C. coli* (Goossens et al., 1986). In this study, growth curves in PB showed good growth of *Campylobacter* for both *C. jejuni* lab strains and for naturally occurring campylobacters on chicken; although in some cases the growth rate or maximum cell number were lower compared to growth in BB and BBc but this was not significant (*P* > 0.05). However, the medium successfully reduced growth of ESBL bacteria in all cases and their numbers dipped below the detection limit within 1–2 h. Levels of *Campylobacter* are yet high enough (6–9.5 log CFU mL^-1^) to produce colonies on the isolation medium if 10 μL is streaked.

Currently, the ISO-protocol for detection of *Campylobacter* is revised (ISO, 2015) and in this protocol, a distinction is made between different food samples, where PB is advised for samples in which high background flora such as ESBL bacteria is suspected. BB is still recommended for samples with low numbers of non-target organisms and low numbers of potentially stressed or sublethally damaged *Campylobacter*. The revision of the ISO-protocol is supported by the results described in this paper and using the new protocol will probably improve *Campylobacter* detection from food samples, provided that labs choose the correct enrichment broths for their specific samples. Furthermore, the advice to use two isolation media with different selective principles will also reduce the risk of overgrowth of *Campylobacter* by non-target flora in at least one of the agars thereby lowering the number of false negative detection results.

Thermotolerant *Campylobacter*, such as *C. jejuni*, cannot grow below 30°C, however, metabolic activity in the cells has been described even at 4°C, showing that not all processes in the cells have stopped (Hazeleger et al., 1998) and cooling and freezing are considered to be stressful for *Campylobacter* in broth (Ray and Johnson, 1984; Jasson et al., 2007) and in raw milk and river water (Humphrey, 1986). Therefore, subsequent growth in detection procedures may be impeded. To overcome cold stress, a pre-enrichment step of 2–4 h at 37°C was described by Humphrey (1986, 1989) in water, milk, and poultry samples to resuscitate sublethally damaged cells at a less selective temperature. In this paper, cooling and freezing, being common factors in the production chain of poultry, were used to induce sublethal damage in the naturally present or inoculated *Campylobacter* on chicken before the advised BB enrichment procedure was started. However, the results do not support the efficacy of a pre-enrichment step, using BB at 37°C prior to incubation at 41.5°C as recommended in the ISO-protocol (ISO, 2006). The different findings may be explained by the use of different enrichment broths and a more selective temperature of 43°C used by Humphrey (1986, 1989). The isolation temperature for *Campylobacter* has since been reduced to 41.5°C for practical reasons, to match with enrichment temperatures for detection of *Salmonella* and *E. coli* O157 (Corry and Atabay, 2012). Taking also into account that changing the incubation temperature from 37 to 41.5°C after 4–6 h of pre-enrichment is troublesome in routine laboratory practice, omission of the pre-enrichment step in the current procedure of enrichment in BB could be considered.

## Author Contributions

WH, WJ-R, and HB contributed to conception and design. WH and HB contributed to acquisition, analysis, and interpretation of data. WH, WJ-R, and HB drafted and/or revised the article.

## Conflict of Interest Statement

The authors declare that the research was conducted in the absence of any commercial or financial relationships that could be construed as a potential conflict of interest.

## Abbreviations

BB: Bolton broth
BBc: Bolton broth supplemented with clavulanic acid
BHI: brain heart infusion broth
CAB: Columbia agar base
CFA: Campyfood agar plates
CFU: colony forming units
ESBLs: extended spectrum β-lactamase producing bacteria
HI: heart infusion broth
ISO: International Organization for Standardization
mCCDA: modified charcoal-cefoperazone-deoxycholate agar
PB: Preston broth
RCA: Rapid’*Campylobacter* agar plates

## References

[B1] Baylis C. L. MacPhee S. Martin K. W. Humphrey T. J. Betts R. P. (2000). Comparison of three enrichment media for the isolation of *Campylobacter* spp. from foods. *J. Appl. Microbiol.* 89 884–891. 10.1046/j.1365-2672.2000.01203.x 11119165

[B2] Bolton F. J. Robertson L. (1982). A selective medium for isolating *Campylobacter jejuni/coli*. *J. Clin. Pathol.* 35 462–467. 10.1136/jcp.35.4.462 7042765 PMC497682

[B3] Bortolaia V. Guardabassi L. Trevisani M. Bisgaard M. Venturi L. Miki Bojesen A. (2010). High diversity of extended-spectrum-lactamases in *Escherichia coli* isolates from Italian broiler flocks. *Antimicrob. Agents Chemother.* 54 1623–1626. 10.1128/AAC.01361-09 20100875 PMC2849389

[B4] Chon J.-W. Kim H. Kim H.-S. Seo K.-H. (2013a). Improvement of modified charcoal-cefoperazone-deoxycholate agar by addition of potassium clavulanate for detecting *Campylobacter* spp. in chicken carcass rinse. *Int. J. Food Microbiol.* 165 7–10. 10.1016/j.ijfoodmicro.2013.04.006 23685466

[B5] Chon J.-W. Kim H. Yim J.-H. Kim M.-S. Park J.-H. Seo K.-H. (2013b). Development of a selective enrichment broth supplemented with bacteriological charcoal and a high concentration of polymyxin B for the detection of *Campylobacter jejuni* and *Campylobacter coli* in chicken carcass rinses. *Int. J. Food Microbiol.* 162 308–310. 10.1016/j.ijfoodmicro.2013.01.018 23474610

[B6] Chong Y. Yoshikiyo I. Kamimura T. (2011). Genetic evolution and clinical impact in extended-spectrum β-lactamase-producing *Escherichia coli* and *Klebsiella pneumoniae*. *Inf. Genet. Evol.* 11 1499–1504. 10.1016/j.meegid.2011.06.001 21689785

[B7] Cohen Stuart J. Van den Munckhof T. Voets G. Scharringa J. Fluit A. Leverstein-Van Hall M. (2012). Comparison of ESBL contamination in organic and conventional retail chicken meat. *Int. J. Food Microbiol.* 154 212–214. 10.1016/j.ijfoodmicro.2011.12.034 22260927

[B8] Corry J. E. L. Atabay H. I. (2012). “Culture media for the isolation of *Campylobacter*s, *Helicobacters* and *Arcobacters*,” in *Handbook of Culture Media for Food and Water Microbiology* 3rd Edn eds Corry J. E. L. Curtis G. D. W. Baird R. M. (Cambridge: Royal Society of Chemistry) 403–450.

[B9] Costa L. Radhouani H. Gomes C. Igrejas G. Poeta P. (2010). High prevalence of extended-spectrum β-lactamases *Escherichia coli* and vancomycin-resistant enterococci isolates from chicken products. A problem of public health. *J. Food Safety* 30 141–153. 10.1111/j.1745-4565.2009.00195.x

[B10] Depoorter P. Persoons D. Uyttendaele M. Butaye P. De Zutter L. Dierick K. (2012). Assessment of human exposure to 3rd generation cephalosporin resistant *E. coli* (CREC) through consumption of broiler meat in Belgium. *Int. J. Food Microbiol.* 159 30–38. 10.1016/j.ijfoodmicro.2012.07.026 22938836

[B11] Dierikx C. van der Goot J. Fabri T. van Essen-Zandbergen A. Smith H. Mevius D. (2013). Extended-spectrum-beta-lactamase- and AmpC-beta-lactamase-producing *Escherichia coli* in dutch broilers and broiler farmers. *J. Antimicrob. Chemother.* 68 60–67. 10.1093/jac/dks349 22949623

[B12] European Food Safety Authority [EFSA] (2011). Analysis of the baseline survey on the prevalence of *Campylobacter* in broiler batches and of *Campylobacter* and *Salmonella* on broiler carcasses in the EU, 2008 – Part A: *Campylobacter* and *Salmonella* prevalence estimates. *EFSA J.* 8:1503 10.2903/j.efsa.2010.1503

[B13] European Food Safety Authority [EFSA] and European Centre for Disease Prevention and Control [ECDC] (2015). The European Union summary report on trends and sources of zoonoses zoonotic agents and food-borne outbreaks in 2014. *EFSA J.* 13:4329 10.2903/j.efsa.2015.4329

[B14] Goossens H. De Boeck M. Coignau H. Vlaes L. Van den Borre C. Butzler J.-P. (1986). Modified selective medium for isolation of *Campylobacter* spp. from feces: comparison with Preston medium, a blood-free medium, and a filtration system. *J. Clin. Microbiol.* 24 840–843. 3771769 10.1128/jcm.24.5.840-843.1986 PMC269038

[B15] Habib I. Uyttendaele M. De Zutter L. (2011). Evaluation of ISO 10272: 2006 standard versus alternative enrichment and plating combinations for enumeration and detection of *Campylobacter* in chicken meat. *Food Microbiol.* 28 1117–1123. 10.1016/j.fm.2011.03.001 21645809

[B16] Hazeleger W. C. Wouters J. A. Rombouts F. M. Abee T. (1998). Physiological activity of *Campylobacter jejuni* far below the minimal growth temperature. *Appl. Env. Microbiol.* 64 3917–3922. 9758819 10.1128/aem.64.10.3917-3922.1998 PMC106578

[B17] Humphrey T. J. (1986). Techniques for the optimum recovery of cold injured *Campylobacter jejuni* from milk or water. *J. Appl. Bacteriol.* 61 125–132. 10.1111/j.1365-2672.1986.tb04265.x 3771411

[B18] Humphrey T. J. (1989). An appraisal of the efficacy of pre-enrichment for the isolation of *Campylobacter jejuni* from water and food. *J. Appl. Bacteriol.* 66 119–126. 10.1111/j.1365-2672.1989.tb02461.x 2708169

[B19] ISO (2006). ISO 10272-1:2006. *Microbiology of Food and Animal Feeding Stuffs – Horizontal Method for Detection and Enumeration of Campylobacter spp. – Part 1: Detection Method*. Geneva: ISO.

[B20] ISO (2015). ISO/DIS 10272-1:2015. *Microbiology of the Food Chain – Horizontal Method for Detection and Enumeration of Campylobacter – Part 1: Detection Method*. Geneva: ISO.

[B21] Jasson V. Sampers I. Botteldoorn N. López-Gálvez F. Baert L. Denayer S. (2009). Characterization of *Escherichia coli* from raw poultry in Belgium and impact on the detection of *Campylobacter jejuni* using Bolton broth. *Int. J. Food Microbiol.* 135 248–253. 10.1016/j.ijfoodmicro.2009.09.007 19786312

[B22] Jasson V. Uyttendaele M. Rajkovic A. Debevere J. (2007). Establishment of procedures provoking sub-lethal injury of *Listeria monocytogenes, Campylobacter jejuni* and *Escherichia coli* O157 to serve method performance testing. *Int. J. Food Microbiol.* 118 241–249. 10.1016/j.ijfoodmicro.2007.07.016 17719670

[B23] Kawamura K. Goto K. Nakane K. Arakawa Y. (2014). Molecular epidemiology of extended-spectrum β-lactamases and *Escherichia coli* isolated from retail foods including chicken meat in Japan. *Foodborne Pathog. Dis.* 11 104–110. 10.1089/fpd.2013.1608 24093132

[B24] Moran L. Kelly C. Cormican M. McGettrick S. Madden R. (2011). Restoring the selectivity of Bolton broth during enrichment for *Campylobacter* spp. from raw chicken. *Lett. Appl. Microbiol.* 52 614–618. 10.1111/j.1472-765X.2011.03046.x 21488911

[B25] Olsen R. H. Bisgaard M. Löhren U. Robineau B. Christensen H. (2014). Extended spectrum β-lactamase-producing *Escherichia coli* isolated from poultry: a review of current problems, illustrated with some laboratory findings. *Avian Pathol.* 43 199–208. 10.1080/03079457.2014.907866 24666286

[B26] Overdevest I. Willemsen I. Rijnsburger M. Eustace A. Xu L. Hawkey P. (2011). Extended-spectrum β-lactamase genes of *Escherichia coli* in chicken meat and humans, the Netherlands. *Emerg. Infect. Dis.* 17 1216–1222. 10.3201/eid1707.110209 21762575 PMC3381403

[B27] Payne D. J. Cramp R. Winstanley D. J. Knowles D. J. (1994). Comparative activities of clavulanic acid, sulbactam, and tazobactam against clinically important betalactamases. *Antimicrob. Agents Chemother.* 38 767–772. 10.1128/AAC.38.4.767 8031044 PMC284540

[B28] Paulsen P. Kanzler P. Hilbert F. Mayrhofer S. Baumgartner S. Smulders F. J. M. (2005). Comparison of three methods for detecting *Campylobacter* spp. in chilled or frozen meat. *Int. J. Food Microbiol.* 103 229–233. 10.1016/j.ijfoodmicro.2004.12.022 15985304

[B29] Ray B. Johnson C. (1984). Sensitivity of cold-stressed *Campylobacter jejuni* to solid and liquid selective environments. *Food Microbiol.* 1 173–176. 10.1016/0740-0020(84)90031-5

[B30] Scotter S. L. Humphrey T. J. Henley A. (1993). Methods for the detection of thermotolerant campylobacters in food: results of an inter-laboratory study. *J. Appl. Bact.* 74 155–163. 10.1111/j.1365-2672.1993.tb03009.x 8444645

[B31] Smith S. Meade J. McGill K. Gibbons J. Bolton D. Whyte P. (2015). Restoring the selectivity of modified charcoal cefoperazone deoxycholate agar for the isolation of *Campylobacter* species using tazobactam, a β-lactamase inhibitor. *Int. J. Food Microbiol.* 210 131–135. 10.1016/j.ijfoodmicro.2015.06.014 26119190

[B32] Ugarte-Ruiz M. Gomez-Barrero S. Porrero M. C. Alvarez J. Garcıa M. Comeron M. C. (2012). Evaluation of four protocols for the detection and isolation of thermophilic *Campylobacter* from different matrices. *J. Appl. Microbiol.* 113 200–208. 10.1111/j.1365-2672.2012.05323.x 22533742

[B33] Uyttendaele M. Debevere J. (1996). Evaluation of Preston medium for detection of *Campylobacter jejuni* in vitro and in artificially and naturally contaminated poultry products. *Food Microbiol.* 13 115–122. 10.1006/fmic.1996.0015

[B34] Zwietering M. H. Jongenburger I. Rombouts F. M. Van ’t Riet K. (1990). Modeling of the bacterial growth curve. *Appl. Env. Microbiol.* 56 1875–1881. 16348228 10.1128/aem.56.6.1875-1881.1990 PMC184525

